# The questions on violence (FOV) tool for interpersonal violence inquiry in Swedish healthcare settings – evaluation of content validity, face validity and test-retest reliability

**DOI:** 10.1186/s12913-024-11708-3

**Published:** 2024-10-16

**Authors:** Solveig Lövestad, Karin Sjöström, Josefin Björk, Karin Örmon

**Affiliations:** 1 Department of Public Health and Community Medicine, Institute of Medicine, Sahlgrenska Academy, University of Gothenburg, Medicinaregatan 18A, Göteborg, 41390 Sweden; 2 The Västra Götaland Region Competence Centre on Intimate Partner Violence (VKV), Kungsgatan 12, Göteborg, 41119 Sweden; 3 Department of Care Science, Faculty of Health and Society, Malmö University, Jan Waldenströms gata 25, Malmö, 214 28 Sweden; 4 Department of Health, Blekinge Institute of Technology, Valhallavägen 10, Karlskrona, 371 79 Sweden

**Keywords:** Reliability, Validity, Instrument, Routine inquiry, Health care, Violence, Interpersonal violence

## Abstract

**Background:**

Previous research indicates that routine inquiry or screening conducted by healthcare providers may significantly increase the identification of interpersonal violence. There is a lack of comprehensive instruments to routinely assess patients about interpersonal violence and violence against children in the household. The purpose of this study was to assess the content validity, face validity and reliability of the Questions on Violence (FOV) tool, an instrument specifically designed for routine inquiries about interpersonal violence in healthcare settings within the Swedish context.

**Methods:**

The content validity, face validity and reliability of the FOV instrument was assessed through (1) a content validity index with six experts in the field of intimate partner violence, (2) cognitive interviews with nine patients recruited from a primary healthcare facility, and (3) an evaluation of the test-retest reliability based on responses from 37(50.0%) university students. The intraclass correlation coefficient, model 2.1, was calculated to assess the degree of correlation and agreement between the two measurements.

**Results:**

Calculations based on the content validity index indicated that five out of seven items had excellent content validity (≥ 0.78). The average content validity index of included items was 0.88, which is slightly below the recommended threshold for excellent content validity. The results based on the cognitive interviews revealed that participants found the seven items to be relevant and easy to understand. Overall, the participants agreed that the concept of ‘close relationships’ primarily encompassed intimate partners, family members, and close friends. The value of the intraclass correlation coefficient was 0.85 (0.77–0.91; CI 95%), indicating good reliability with an interval of good to excellent test-retest reliability.

**Conclusions:**

The results demonstrate that the seven-item FOV instrument has good content and face validity as well as good to excellent test-retest reliability. The current study provides healthcare professionals with a short yet comprehensive instrument for identifying patients who have experienced or perpetrated different forms of interpersonal violence.

**Supplementary Information:**

The online version contains supplementary material available at 10.1186/s12913-024-11708-3.

## Background

Interpersonal violence is a global public health challenge that has significant adverse effects on health and society [1]. It encompasses various types of violence, including physical, sexual, psychological, and economic violence, as well as controlling behaviour and neglect [2]. The World Health Organization (WHO) groups interpersonal violence into two main categories: family and intimate partner violence (FIPV) and community violence (CV) [2]. FIPV involves violence against family members, such as intimate partners, children, and elderly individuals, often occurring within the home. In Sweden, FIPV is commonly defined as ‘violence in close relationships’ (‘våld i nära relationer’ in Swedish) [3]. CV, on the other hand, refers to violence between unrelated individuals, whether acquainted or not, usually happening outside the home [2]. CV encompasses youth violence, random acts of violence, sexual assault or rape committed by strangers, and violence occurring within institutional settings such as schools, workplaces, and healthcare facilities [2].

Intimate partner violence (IPV), which falls into the category of FIPV, is perpetrated by a current or former partner and is one of the most common forms of violence against women [2]. Approximately one in three women worldwide have experienced physical and/or sexual violence by an intimate partner at some time in their lives [1]. Men, in contrast, are more likely to experience CV, i.e., violence from strangers that occurs outside their home [2]. Violence and abuse against children include all types of violence targeting individuals under the age of 18, regardless of whether the perpetrator is a parent, caregiver, peer, or stranger [4]. Global estimates indicate that approximately three out of four children (approximately 300 million children) between the ages of two and four years, experience frequent exposure to physical punishment and/or psychological violence from a parent or caregiver [4]. Furthermore, one in four children under the age of five live with a mother who is a victim of IPV [4]. Children who experience violence and abuse are at a higher risk of facing negative health and social consequences. These include cognitive dysfunctions, depression, somatic symptoms, poor academic performance, and engagement in risky behaviours [5]. Furthermore, exposure to violence and witnessing (having seen and/or heard) IPV during childhood can lead to physical and mental health problems in adulthood [6–8]. Moreover, exposure to violence during childhood increases the likelihood of men and women becoming perpetrators of child abuse, of men becoming perpetrators of IPV, and of women becoming victims of IPV [4, 9]. Therefore, efforts aimed at preventing violence against children and IPV are crucial, as they may contribute to reducing and preventing both forms of violence [4].

The existing body of research shows a clear association between mental health problems and IPV perpetration, particularly among men. Symptoms of psychological distress [10], alcohol and drug abuse [11], and depression and anxiety [11], have been identified as factors linked to IPV perpetration. Furthermore, research suggests that experiencing clinically significant levels of mental health problems, such as symptoms of depression, anxiety, and posttraumatic stress syndromes, is associated with the co-occurrence of perpetration of IPV and child abuse [12]. Previous studies show that individuals who perpetrate IPV may seek help from healthcare providers due to various factors, including mental health issues, alcohol and substance abuse problems [13, 14], and relationship difficulties, e.g., issues with anger management [14]. Thus, it is reasonable to assume that perpetrators of violence against children may seek help for similar reasons. Victims of IPV, on the other hand, may seek assistance for injuries and other negative health consequences resulting from the violence they have been subjected to [2]. Given these circumstances, healthcare providers play a pivotal role in identifying patients who are experiencing IPV [15, 16] and/or cases of child abuse [5], as well as patients who may be at risk of perpetrating IPV [17] and/or child abuse.

Previous research indicates that routine inquiry or screening conducted by healthcare providers may significantly increase the identification of interpersonal violence, such as IPV [17–22] and child abuse [23]. Screening refers to systematic assessments conducted across entire population groups to detect exposure to violence [18], while routine inquiry involves consistent questioning in specific settings or when indicators of abuse are present [24]. In a recent study conducted in the US involving 155 male and female patients screened during routine mental health care visits, 49% disclosed exposure to IPV and 46.5% disclosed perpetrating IPV [21]. Additionally, prior studies indicate that patients generally consider it positive to be asked about violence within the health care setting [25–27]. However, screening or routine inquiry alone is not sufficient for health care personnel to identify those exposed to or perpetrating violence. Prior studies show that health care professionals face several barriers to asking about violence, including a lack of training, education and the absence of referral options [20, 28]. Therefore, screening or routine inquiry should be supported by prior and recurring training, management support and clear guidelines on documenting and responding to victims and perpetrators of violence [20, 29, 30].

To ensure an accurate identification of patients at risk, it is important to use valid and reliable tools when screening or routinely assessing patients for experiences of interpersonal violence [16, 31]. In addition, screening tools should be brief and easily understandable to facilitate their practical use [32], as overly time-consuming or extensive instruments may impede their practical implementation. Furthermore, it is crucial that screening instruments include items that capture different types of violence, as this enhances their comprehensiveness and effectiveness in identifying victims and perpetrators of IPV [31].

In 2021, the Swedish government introduced a new legislation criminalising adults who subject children to witnessing violence between family members and/or those to whom the child has a strong attachment in everyday life. The legislation requires that the healthcare staff consider the child’s need for information, advice, and support in cases where the child is exposed to violence, including cases of witnessing IPV [33]. Therefore, it is essential that healthcare providers in Sweden assess patients not only for IPV but also for potential violence and abuse against children in their families. To date, there is a lack of screening instruments that adequately address different forms of interpersonal violence as well as including questions about potential violence against children in the household [32, 34]. The Hurt Insult Threat Scream (HITS) tool, originally designed to detect intimate partner violence in women, was adapted in 2016 to screen for interpersonal violence more broadly, including violence involving family members and friends [35]. However, the HITS- tool does not include any question about exposure to sexual violence or violence against children in the household [35]. To our knowledge, there is only one previous instrument that includes a question where the respondent is asked about violence against children in the household [32, 36]. To address this gap, the Questions on Violence tool, abbreviated as FOV (‘Frågor om våld’ in Swedish), was developed with the aim of encompassing different forms of interpersonal violence while at the same time maintaining brevity by minimising the number of questions [37]. The inclusion of comprehensive questions about exposure to and perpetration of interpersonal violence in routine assessments, is important for healthcare providers in order to identify patients who experience poly-victimisation by multiple perpetrators. Specific circumstances of violence exposure, such as sexual assault by a stranger or ongoing violence within an intimate relationship, may require individual and tailored support for victims [37]. Furthermore, previous research highlights the need for preventive efforts that address different types of interpersonal violence simultaneously [38]. Thus, implementing routine inquiry with instruments that include comprehensive items about different forms of interpersonal violence may enable healthcare providers to identify patients who perpetrate and/or experience multiple forms of violence and are victimised by various perpetrators.

When developing a new instrument, it is important that the instrument measures what it is supposed to measure and that it measures in the same way on repeated occasions. Validity and reliability are key aspects that need to be evaluated before the instrument can be used for clinical decision-making or research purposes [39]. Content validity refers to the extent to which the items in an instrument adequately capture the theoretical content domain of a construct [40], while face validity assesses whether the target population perceives the items as clear, relevant, and easy to answer [40]. Content validity is typically assessed by expert judges with extensive knowledge of the subject matter, who evaluate how well each item reflects the conceptual definition of the construct [40, 41]. Face validity can be assessed using either quantitative or qualitative methods, where the target population is interviewed or surveyed to provide their thoughts on each item [40]. Reliability refers to the consistency of a measure when repeated under identical conditions and may be assessed by test-retest reliability which evaluates the consistency of respondents’ sum scores over time [41].

The aim of this study was therefore to evaluate the content validity, face validity and reliability of the FOV tool, an instrument developed for routine inquiry on interpersonal violence in healthcare settings in a Swedish context.

## Methods

### The initial development of the FOV instrument

The development of the FOV tool was implemented in continuous collaboration with staff at the Västra Götaland Region Competence Centre on Intimate Partner Violence, called the VKV (‘Västra Götalandsregionens kompetenscentrum om våld i nära relationer’ in Swedish), and staff from different healthcare settings in the Västra Götaland Region (VGR) [42]. Between 2012 and 2014, the VKV, in collaboration with different healthcare providers in the VGR, implemented a project with the purpose of informing and educating the healthcare staff about established guidelines and procedures for routine inquiry on IPV. A specific aim of the project was to address violence against children in the household [43]. In this project, the healthcare staff used the three-item Partner Violence Screen (PVS) to assess IPV [44] and one additional item to inquire about violence against children in the family. In the subsequent evaluation, based on focus group interviews with the healthcare staff, the results showed that the use of the PVS had shortcomings since it lacked questions about psychological and sexual violence [43]. The healthcare staff further mentioned that the PVS, despite its brevity, was hard to understand [43]. The evaluation concluded that there was a need to develop a brief and easily understandable instrument that would address different forms of violence within a Swedish context [43]. As a result, the staff at the VKV examined the existing literature available at that time. Apart from the PVS, other common IPV screening tools included the five-item Abuse Assessment Screen (AAS) [45], the four-item Hurt, Insult, Threatened or Screamed (HITS) questionnaire [46], the four-item Humiliation, Afraid, Rape and Kick (HARK) [47], the eight-item Woman Abuse Screening Tool (WAST) [48], the three-item STaT screen [49], and the 10-item Women´s Experience with Battering (WEB) questionnaire [50]. Among these instruments, the HITS [46], AAS [45], HARK [47], WAST [48] and STaT [49] included items that asked about exposure to psychological violence, such as whether the respondent had been insulted, talked down to, humiliated, emotionally abused or threatened by a partner or someone else [45–49]. Questions regarding exposure to physical violence were included in all instruments except for the WEB questionnaire [50]. These questions inquired whether the respondent had been hit, slapped, kicked, pushed, punched and/or ‘otherwise physically hurt’ by a partner or someone else [45–49]. Among these instruments only the AAS, WAST and HARK included questions on sexual violence, asking if the respondent had been raped, ‘forced to have sexual activities’, or ‘abused sexually’ [45, 47, 48]. In addition to assessing abuse by an intimate partner, both the AAS [45] and PVS [44], included questions about abuse by others (‘someone important to you’, ‘someone’, ‘anyone’ and ‘your partner or anyone’), whereas the other instruments focused solely on abuse by a current or former intimate partner [46–50]. Instruments, such as HITS and WAST, offered 3–5 response options, which may have affected the time required for routine inquiry [46, 48]. Another instrument, the 13-item NorVold Abuse Questionnaire (NorAQ), validated in a Swedish healthcare context, measures mild, moderate, and severe emotional, physical, and sexual abuse by various perpetrators [51]. Additionally, two previous population-based studies of men [52] and women [53], examined the psychometric properties of the Swedish version of the WHO Violence Against Women Instrument (WAVI), which demonstrated good construct validity and internal reliability for the female population [53]. The Swedish version of WAVI includes psychological, physical and sexual IPV with in total 13 items (four, six and three items respectively) [52]. However, none of the instruments, fulfilled the requirements of being brief, including exposure to violence by individuals other than an intimate partner, addressing violence against children in the household, and inquiring about the respondent’s own perpetration of violence. Thus, based on examined instruments, including the Swedish version of the WAVI [53], the staff at the VKV, in collaboration with healthcare staff in the VGR, developed the FOV tool.

In 2013, the FOV tool was employed for the first time in a project including healthcare staff from different rehabilitation centres [37]. The results, based on the subsequent evaluation of the project, showed that the FOV tool facilitated routine inquiry about interpersonal violence in healthcare settings and increased the healthcare staff’s ability to identify victims and perpetrators of interpersonal violence [37]. Since then, and in continuous discussions with different healthcare providers, the FOV tool has undergone minor changes to simplify its structure and practical use. The 2019 version of the FOV tool, comprising the set of seven items, was validated for the present study (Table 1). The 2019 version of the FOV tool assesses three domains of violence: psychological/emotional, physical, and sexual. Items 1–3 and 5 assess FIPV by asking about violence exposure by ‘someone close to you’. This is also true for item 7, where the patient is asked whether children in the household have been exposed to any of the three forms of violence by someone close to the child. Item 4 assesses CV by asking about violence exposure by ‘someone who is *not* close to you’, whereas item 6 assesses both FIPV and CV, as the question is framed more broadly, asking whether the respondent has ‘subjected someone else to violence’.

**Table 1:** Vignette and items in the ‘Questions about violence’ (FOV) instrument

**Vignette**	Violence in close relationships affects how you feel both physically and mentally, which is why we ask about violence. It also affects the physical and mental health of your children. Children have the right to grow up without violence. Violence includes being victimised yourself or seeing someone else being victimised. You can get support if you have been a victim of violence or if you have seen or heard violence against someone close to you during your childhood. You can also get support if you have perpetrated violence yourself. *________________________________* By ‘someone close to you’ we mean a partner, a family member, a relative, or another important person, such as a close friend.
**Items**	
1	Have you been threatened, controlled, humiliated, harassed, or similar, by someone close to you?
2	Have you been held against your will, pushed, punched, kicked, or otherwise injured, by someone close to you?
3	Have you felt pressured or coerced into sexual acts by someone close to you?
4	Have you been subjected to violence, as described in questions 1–3, by someone who is not close to you?
5	When growing up, did you see or hear someone close to you being subjected to violence as described in questions 1–3?
6	As an adult, have you subjected someone else to violence as described in questions 1–3?
7	Have any children you live with, or have lived with, been subjected to violence as described in questions 1–3?

### Assessing the content validity, face validity and reliability of the FOV

**Fig. 1:**
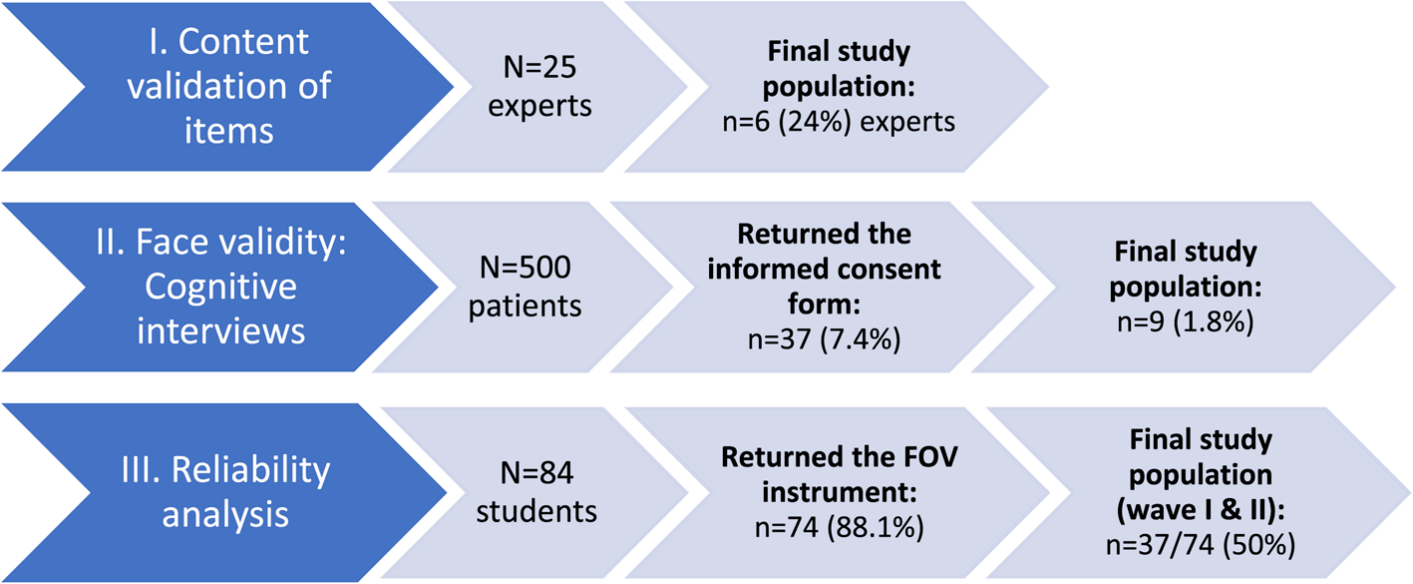
Evaluation process (I-III) of the validity and reliability of the FOV instrument

The evaluation of the content validity, face validity and reliability of the FOV instrument was performed in three different parts (described in more detail below): (1) the content validity index (CVI) with experts in the field, (2) cognitive interviews (CI) with the target population to enhance the face validity of the instrument, and (3) the evaluation of the test-retest reliability to measure the consistency and stability of the instrument (Fig. 1). The three parts were not carried out in sequence, i.e., part one and part two were carried out in parallel, whereas part three was carried out last.

#### Part I: the content validation of items

Content validity refers to the extent to which a collection of items in a sample effectively represents a comprehensive operational definition of a particular construct [54]. The CVI is widely used as an index of interrater agreement concerning the relevance of an item [39]. It involves a carefully selected group of experts who evaluate the relevance of each item in an instrument, with the purpose of ensuring that the questions accurately reflect what is going to be measured [39]. It is recommended that a minimum of three, but no more than ten, experts rate the relevance of each item [55]. A CVI value is calculated for each item on a scale (I-CVI), or for the overall scale (S-CVI) [39]. The items are commonly rated on a 4-point scale, where a score of 1= ‘not relevant’, a score of 2 = ‘somewhat relevant’, a score of 3 = ‘quite relevant’, and a score of 4 = ‘highly relevant’ [39]. A minimum recommended I-CVI value of 0.78 indicates a good content validity of an item [39]. The S-CVI can be calculated through the Scale Content Validity Index/Universal Agreement (S-CVI/UA) method or the Scale Content Validity Index-Average (S-CVI/Ave) method. The S-CVI/UA method calculates the proportion of items that all content experts have rated as ‘quite’ or ‘highly relevant’, whereas the S-CVI/Ave is used to calculate the I-CVI for each item, followed by computing the average I-CVI across all the items [54]. The use of the S-CVI/UA may significantly impact the S-CVI value, since the likelihood that the experts achieve complete agreement at the item level (I-CVI = 1.0) decreases as the number of experts increases, consequently leading to the probability of achieving a low S-CVI [54]. Polit, Beck and Owen [39] recommend an S-CVI/Ave of 0.90 or higher, including complete agreement (I-CVI = 1.00) on some items in the scale and a few items with an I-CVI of at least 0.78.

##### Study population and data collection

A purposive sampling strategy was conducted in 2021 to identify researchers and professionals with substantial experience within the field of IPV [42]. Through different webpages of universities and IPV competence centres in Sweden, 25 experts (19 researchers and six professionals) were identified and invited by digital mail to participate in the study (Fig. 1). The first six answers received were from persons eligible for the study. The participants were informed through digital mail about the purpose of the study and how to conduct the CVI [42]. The digital mail also contained a form for the CVI registration, and that if the informant decided to send their registered CVI responses to the research team, this would be regarded as their consent to participate in the validation study. Thus, six experts consented to participate, consisting of five researchers from different universities and one professional from an IPV competence centre [42].

##### Data analysis

In line with previous research [39], the response alternatives ‘quite relevant’ (= 3), ‘highly relevant’ (= 4), ‘not relevant’ (= 1), and ‘somewhat relevant’ (= 2) were dichotomised into ‘relevant’ (responses 3 and 4) and ‘not relevant’ (responses 1 and 2) prior to calculation of the index [42]. The I-CVI was calculated for each item as the proportion of expert reviewers rating the item as 3 or 4 (‘quite relevant’ or ‘highly relevant’), divided by the total number of experts who evaluated the FOV screening instrument. To calculate the S-CVI/UA, the total number of items rated as 3 or 4 by all six experts was divided by the total number of items in the instrument (= 7) [42]. To calculate the S-CVI/AVE, the sum of the I-CVI was divided by the total number of items.

#### Part II: face validity

CIs were conducted to evaluate the clarity and comprehensibility of the items in the FOV tool. CI is a qualitative interview method that is often used in the validation of questionnaires to examine how the participants interpret the questions. CIs also provide indications as to whether revisions are necessary to perform [56–58]. The cognitive process involved when answering a questionnaire consists, according to Tourangeau [59], of four steps: interpreting the question, recalling information from the memory to be able to answer, formulating an assessment, and, finally, choosing an appropriate response option [59, 60]. There are two different strategies for conducting a CI for validation purposes: the ‘think-aloud’ and the probing strategies [61]. In the ‘think-aloud’ strategy, the participant is asked to explain what he/she thinks when reading the question aloud, whereas in the probing strategy, the interviewer asks follow-up questions (after the respondent has thought out aloud) about the participant’s own thoughts when answering the question. This gives insight into the respondent’s cognitive process as they answer the questions, which in turn may be used for the revision of the instruments and for the improvement of the validity and reliability of the questionnaire [61–63].

##### Study population and data collection

Participants were recruited through a primary healthcare centre in Gothenburg, the second largest city in Sweden [42]. All patients seeking health care (*n* = 500) at the primary healthcare facility received an envelope containing a prepaid envelope, information about the research project and the aim of the study, the FOV questionnaire, and a written informed consent form (Fig. 1). Patients were instructed not to fill out the FOV questionnaire, as it was enclosed with the informational letter only to inform patients about the content and wording of the questions [42]. Patients were eligible if they were 18 years or older, had no cognitive problems, and were able to read and speak Swedish. Interested patients were asked to submit their written informed consent to the research group [42].

A total of 37 patients (7.4%) returned the signed informed consent form [42]. These individuals were subsequently contacted through mail or telephone by the third author. Seven individuals withdrew their participation upon contact, 19 individuals did not respond to the repeated contact attempts, and two individuals withdrew their participation after having misinterpreted the focus of the study. The final study population consisted of nine (1.8%) participants: eight women and one man, aged 31 to 75 years [42]. The participants’ experiences of interpersonal violence varied, ranging from no experience at all to having experienced such violence to a significant extent. The time and place for the interviews were agreed upon with the third author and in collaboration with the participants. The third author conducted the CIs and used a semi-structured interview guide to ensure the comprehensibility and interpretation of each item included in the FOV instrument (Supplementary file). Six out of nine interviews were conducted digitally, and three interviews were conducted face to face. The interviews lasted between 20 and 120 min. The ‘think-aloud’ method with probing was used to stimulate the participants to develop their thoughts when needed [42]. Considering the sensitive nature of the subject (interpersonal violence), spontaneous stories and personal experiences shared by the participants were recognised and given ample room for expression. All participants were provided with contact information for a counsellor in case they required support afterwards [42].

##### Data analysis

All interviews were audio-recorded and manually transcribed [42]. The answers to and comments on each item were extracted from the interviews and manually inserted into a table overview of the questions. A qualitative content analysis according to Burnard (1996) [64] was performed for each question separately, deriving common themes from the different interviews by focusing on the respondents’ understanding and interpretation of the question as well as possible aspects that made interpretation difficult. The derived themes were then reviewed by the researchers together, regarding possible changes of the formulation of the questions, in order to further enhance the understanding and interpretation of them.

#### Part III: reliability analysis

The test-retest reliability measures the consistency and stability of a questionnaire administered to the same participant on two different occasions [65]. The test-retest procedure not only reflects the degree of correlation but also the agreement between measurements. Mathematically, reliability represents a ratio of true variance over true variance plus error variance [66, 67]. The reliability value ranges between 0 and 1, with values closer to 1 representing stronger reliability. Previously, the Pearson correlation coefficient, the paired t test, and the Bland‒Altman plot were used to calculate test-retest reliability. However, the paired t test and the Bland‒Altman plot are methods for analysing agreement between groups, whereas the Pearson correlation coefficient is only a measure of correlation; hence, they are all considered nonideal measures of reliability [68]. A more desirable measure of reliability should reflect both the degree of correlation and the agreement between measurements. The intraclass correlation coefficient (ICC) is such an index [68]. It describes how strongly units in the same group resemble each other. The ICC 2.1 method, meaning a 2-way mixed-effects model, single measurement (i.e., one measurement for stability) and absolute agreement, is the model suggested for test-retest reliability studies [68]. While it is viewed as a type of correlation, unlike most other correlation measures it operates on data structured as groups rather than data structured as paired observations. ICC values below 0.5 indicate poor reliability, values falling between 0.5 and 0.75 indicate moderate reliability, values within the range of 0.75 and 0.9 indicate good reliability, and values exceeding 0.90 indicate excellent reliability [68].

##### Study population and data collection

Students enrolled in semesters three and six in a three-year bachelor’s programme at a university in Sweden were verbally informed about the aim of the study and the procedure of the data collection. To be included, the students had to be 18 years or older, without cognitive problems, and able to read Swedish. Students interested in participating (*n* = 84) in the study received an envelope containing a prepaid envelope, the FOV questionnaire, written information about the purpose of the study, and a written informed consent form for further participation (Fig. 1). Interested students were informed that the study included answering the questionnaire on two different occasions and that they should not disclose whether they had been subjected to additional violence between waves one and two of the study. A total of 74 students (88.1%) returned the self-administered FOV instrument. Within a two-week period, a second envelope containing the same information (except for the written informed consent form) was sent to the same respondents. Of the 74 students who responded to the questionnaire in the first wave, 37 students (50.0%) also responded to the questionnaire in the second wave.

##### Data analysis

The ICC and the 95% confidence intervals was analysed using the Statistical Package for the Social Sciences (SPSS), version 28. The ICC was calculated with a two-way mixed, single measurement analysis (2,1), which is recommended for test-retest reliability according to [68]. An agreement analysis of each question (1–7) in the test-retest analysis was made by calculating the kappa *(k)* value. The *k* agreement measure states how much better than chance the agreement was between the test-retest values for each question in the FOV. The value of *k* ranges from 0 to 1.0, i.e., from no agreement to perfect agreement. The guidelines for kappa were slightly adapted from Landis and Koch (1977) [69], where < 0.20 is poor agreement, 0.21–0.40 fair, 0.41–0.60 moderate, 0.61–0.80 good, and 0.81–1.00 very good agreement. In addition, the mean kappa value for all questions was calculated [69].

## Results

### Content validity results

The I-CVI scores ranged from 0.66 to 1.00 (Table 2). All six experts unanimously agreed on the rating of ‘quite’ or ‘highly relevant’ for four items (1, 2, 3, and 5) out of the seven items (I-CVI = 1.00) in the FOV tool, whereas item 4 was rated as ‘quite’ or ‘highly relevant’ by five out of six experts (I-CVI = 0.83), indicating excellent content validity of the first five items (I-CVI = ≥ 0.78). Item numbers 6 and 7 had an I-CVI of 0.66, indicating fair content validity. The six experts all agreed that four out of the seven items in the instrument were content valid (S-CVI/UA = 0.57). The S-CVI/AVE was 0.88, indicating a S-CVI/AVE slightly below the threshold for excellent content validity (SCVI/AVE = ≥ 0.90).

**Table 2:** Expert rating on the 7-item FOV, total number of expert agreement (n), and item CIVI

Items	Expert 1	Expert 2	Expert 3	Expert 4	Expert 5	Expert 6	Agreement Total (*n*)	Item CVI
1	x	x	x	x	x	x	6	1.00
2	x	x	x	x	x	x	6	1.00
3	x	x	x	x	x	x	6	1.00
4	x	x	x	x	x	-	5	0.83
5	x	x	x	x	x	x	6	1.00
6	x	x	x	x	-	-	4	0.66
7	x	x	x	x	-	-	4	0.66
**Proportion rated as relevant**	1.00	1.00	1.00	1.00	0.71	0.57	**Sum of scores**	
							S-CVI/UA = 0.57	
							S-CVI/AVE = 0.88	

*Item CVI *Item-level content validity index,  *S-CVI/UA *Scale-level content validity index, universal agreement calculation method, *S-CVI/AVE *Scale-level content validity index, average of the I-CVIs for all items in the scale calculation method

(x) = rated as ‘relevant’; (-) = rated as ‘not relevant’

### Results based on the cognitive interviews

Overall, participants found items 1–5 to be relevant and easily comprehensible (Table 1). Many respondents related these items, particularly items 1 and 2 addressing psychological and physical violence, to their personal experiences of violence. The following quote exemplifies this connection: *‘… um… Yes*,* what can I say*,* yes*,* I have been [subjected to violence]*,* and now when I am to think out aloud*,* I think about it directly*,* like. It becomes… well*,* it evokes memories then*,* it does. […] it goes straight to the subject.’*

The participants perceived item 6 as an important question and the concepts and words used in the question as clearly formulated and easy to understand. Some participants, however, argued that it was a question that was difficult to answer not because it was difficult to understand but rather because the question led to reflections about one’s own behaviour and how it was perceived by others. Some of the participants mentioned that it could be difficult to recognise that you have subjected someone to violence, as exemplified by this quote:

[…] of course, one has pushed someone or something like that, when being a bit angry, without it truly being… and one has apologised and such. Probably… a lot of people have done it in some way, and then maybe it hasn’t had any serious consequences. But, well, one would probably answer yes, or I would probably have answered yes to it, even though I don’t have, [don’t] think that I have exposed anyone to violence in close relationships in the way I think of violence in close relationships.

Overall, the participants thought that the concepts and words used in item 7 were clearly formulated and easy to understand, although item 7 was read a couple of times by some of the informants. Those participants who had never lived with children in the home dismissed the question as less relevant to them, while respondents who lived or had lived with children reasoned that the question clearly referred to exposure to violence against children living in the participants’ household or children who had lived in the home before.

Finally, the participants discussed how they interpreted the concept of ‘close relationships.’ A few of the participants interpreted the concept as including only intimate partners, whereas some interpreted the concept more broadly, including both friends at school and healthcare staff. Overall, the participants agreed that they interpreted the concept of ‘close relationships’ as primarily encompassing intimate partners, family members, and close friends.

Based on results from the CIs, which did not include any suggestions for major changes of content or wording in the items, the items were kept intact but corrected according to Swedish grammar with added commas where needed.

#### Results on reliability

The result based on the ICC (2.1) was 0.85 (0.77–0.91; CI 95%), indicating good reliability with an interval of good to excellent reliability; hence, the results were concluded to be valid. When each item of the instrument was examined by the kappa measurement of agreement, we found a 100% test-retest agreement for question 7 and the lowest agreement values for questions 3 and 6 (0.60 and 0.65, respectively) (Table 3).

**Table 3:** Test-retest results for each question based on the kappa agreement *N* = 37

Item number	Question	Kappa values	Agreement
1	Have you been threatened, controlled, humiliated, harassed, or similar, by someone close to you?	0.86	Very good
2	Have you been held against your will, pushed, punched, kicked, or otherwise injured, by someone close to you?	0.80	Good
3	Have you felt pressured or coerced into sexual acts by someone close to you?	0.60	Moderate
4	Have you been subjected to violence, as described in questions 1–3, by someone who is not close to you?	0.72	Good
5	When growing up, did you see or hear someone close to you being subjected to violence as described in questions 1–3?	0.80	Good
6	As an adult, have you subjected someone else to violence as described in questions 1–3?	0.65	Good
7	Have any children you live with, or have lived with, been subjected to violence as described in questions 1–3?	1.00	Very good

## Discussion

The present study evaluated the content validity, face validity and reliability of the ‘Questions on Violence’ (FOV) tool, a new instrument developed for routine inquiry in healthcare settings in a Swedish context. The study was performed in three different parts: the assessment of the CVI with experts in the field to determine the content validity, the use of CIs as a qualitative approach to capture face validity, i.e., the respondents’ interpretation of the items, and the evaluation of the test-retest reliability to measure the consistency and stability of the FOV instrument.

In the content validity process, five out of seven items showed excellent content validity, which is in accordance with the recommendations by Polit and Beck [54]. Of the seven items, two items (items 6–7) had I-CVI scores below the suggested cut-off score of 0.78. According to Polit and colleagues [39], I-CVI scores that are below the suggested cut-off score should be either removed or revised. However, Terwee and colleagues [70] suggest that in the development of an instrument, it is important that the items accurately reflect the areas that are of importance for the target population under study. The target population is the end users of the instrument and can best provide insight into whether a question is understandable. To achieve this, involving the target population in the item selection process becomes a crucial step [70]. The authors further suggest that item reduction techniques do not necessarily guarantee an improvement in content validity [70]. Instead, they propose that focusing on a comprehensive set of items, without reducing their number, can be equally effective [70]. Since the participants in the CIs perceived both item 6 and item 7 as relevant and comprehensible, which indicates good content validity, the two items with an I-CVI score below the suggested cut-off score were kept. The results showed that item 6 (‘As an adult, have you subjected someone else to violence as described in questions 1–3?’) triggered the informants to reflect on their own behaviour, on what constitutes ‘violence’, and on how others may have perceived their behaviour. Engaging in such reflections may serve as a good starting point for a deeper understanding of what constitutes violence and may lead to questioning one’s own behaviour. To effectively prevent interpersonal violence, a supportive infrastructure is crucial [15]. However, long-term sustainable change also requires that individuals become agents of change of their own behaviour [15].

In the CIs, the concept of ‘close relationships’ caused reflections and comments. All participants agreed that the concept included relationships perceived as ‘close’, but the opinion on who is included in the concept of ‘close relationship’ varied to some extent. Since there is no concept in the Swedish language equivalent to ‘interpersonal violence’, and six out of the seven items in the FOV focus on experiences of violence within ‘close relationships’ (i.e., FIPV including close friends), we chose to keep the concept of ‘close relationships’, and the related phrase ‘someone close to you’ (‘en närstående’ in Swedish) in the items and in the vignette. Furthermore, in relation to the vignette in the FOV instrument, the definition of ‘someone close to you’ is described as including others than just family members. Thus, we believe that any confusion or misinterpretation of the concept can be clarified by the vignette and the explanation done by healthcare staff using the FOV when routinely assessing patients about violence. The results based on the single round of CIs in this study did not suggest any major changes to the items in the FOV tool; thus, it was judged that potential problems had been satisfactorily detected and addressed. Theoretically, an instrument can be tested many times until major obstacles and problems have been detected and addressed [71]. However, practical constraints, such as economic and human resources as well as a limited time frame, influence the number of times it is possible to do a test [71]. Additionally, a questionnaire can be tested an infinite number of times and still not be perfect [71]. Overall, the S-CVI/AVE was 0.88, which is slightly below the recommended threshold for excellent content validity [39, 54]. In summary, the results based on the CVIs and CIs suggest that the seven-item FOV scale can be considered to have overall good content and face validity. In addition, the results based on the ICC (1.2) demonstrated that the FOV instrument had good to excellent test-retest reliability, with participants reporting similar answers across the two measurements.

Although our sample size in the reliability analysis was relatively small (*n* = 37), it aligns with the recommendations made by Bujang and colleagues (2024) [72] for analysing the reliability of a questionnaire. The authors recommend that a minimum sample size of 30 respondents is generally sufficient [72]. However, larger sample sizes are suggested for studies assessing construct validity or conducting sensitivity and specificity analyses, especially for questionnaires designed for screening purposes [72]. Therefore, future validation studies should aim for a larger sample size to specify the sensitivity and specificity of the FOV tool.

The main reason for the development of the FOV instrument was the lack of previous instruments for routinely assessing adults visiting healthcare settings regarding both exposure to and perpetration of different forms of interpersonal violence and violence against children in the household [32]. However, for an effective identification of patients involved in violence or exposed to it, screening instruments must be accompanied by healthcare providers who are adequately trained in sensitively asking about violence, who are given ample time and space to engage in these discussions, and who demonstrate sensitivity towards the patients [27]. Furthermore, it is important that established referral options are in place [27].

This study represents the initial phases of the validation process for the FOV tool. Future studies should include tests of dimensionality to determine whether the measurement of items and their functions in the FOV tool, remain consistent across two independent samples or within the same sample at different time points [41]. Additionally, future studies should focus on establishing the tool´s convergent and discriminant validity to assess the extent to which the FOV construct correlates with measures of other constructs, and to determine whether the FOV tool´s measures are novel and not reflective of other constructs [41].

### Limitations

The FOV instrument was validated by assessing content and face validity, as well as reliability in three different subpopulations in Sweden (experts within the field of IPV, patients recruited from a primary healthcare centre, and a group of university students) and may therefore not be generalisable to other populations or settings. Furthermore, the FOV tool was developed for use in routine inquiry in healthcare settings; thus, its applicability for other purposes, such as research purposes in population-based studies, needs to be further explored in future studies. Future research should validate the FOV tool in the population, language, and cultural setting where it is going to be used.

An important limitation in this study is that item 5 (*When growing up*,* did you see or hear someone close to you being subjected to violence as described in questions 1–3*?) relies on the respondents retrospective memories of potential childhood adversities, which in turn may be subject to recall bias [73]. Accurate retrospective recall may be influenced by several factors, including the respondent’s age at the time of exposure to violence, their current mental health and the natural process of forgetting, especially when recalling events that occurred many years earlier [73]. However, previous research has found that prospective reports of adverse childhood experiences confirm associations with adverse childhood experiences retrospectively recalled in adulthood [74, 75], suggesting that completely dismissing or rejecting adults’ retrospective reports of abuse is not justified or appropriate [74].

A potential limitation in this study is that the recruitment of participants for the CIs took place during the COVID-19 pandemic. A general suggestion is that the CIs consist of a range of 5 to 15 interviews [76]. The initial plan was to conduct fifteen face-to-face CIs. However, due to the restrictions and safety measures in place during the spring and summer of 2021, we had to conduct the CIs digitally. Unfortunately, this digital format led to a drop-out of individuals who lacked access to the digital devices required for conducting the interviews. This may have influenced the results, possibly leading to less variability in reflections, interpretations, and answers concerning the items.

Another limitation, which is possibly also a result of the low number of participants in the CIs, is that only one out of the nine participants in the CIs was male. Interpersonal violence does not occur in a social vacuum but arises from socially constructed gender norms [38]. From an early age, social expectations and institutional practices reinforce and normalise physical strength, toughness, and violence among boys and men [38]. Thus, the understanding and interpretation of exposure to and perpetration of violence may vary between men and women due to differences both in individual experiences and in the social roles that shape their perspectives. Future studies should try to include more men when validating the FOV tool in other contexts and settings.

## Conclusions

The aim of the present study was to evaluate the content validity, face validity and reliability of the FOV tool, an instrument developed for routine inquiry on interpersonal violence in healthcare settings in a Swedish context. The results demonstrate that the seven-item FOV instrument has good content and face validity as well as good to excellent test-retest reliability. The current study provides healthcare professionals with a short yet comprehensive instrument for identifying patients who have experienced or perpetrated different forms of interpersonal violence. However, considering that the FOV tool was designed for routine inquiry in healthcare settings within a Swedish context, future research should confirm the validity of the FOV tool in the specific population, language, and cultural setting where it is intended to be employed.

## Electronic supplementary material

Below is the link to the electronic supplementary material.

Supplementary Material 1.

## Data Availability

The deidentified data generated and analysed during the current study may be available from the corresponding author upon reasonable request.

## Abbreviations

WHO: World Health Organization
FIPV: Family and intimate partner violence
CV: Community violence
IPV: Intimate partner violence
FOV: Frågor om våld
VKV: Västra Götaland Region Competence centre on intimate partner violence
VGR: Västra Götaland Region
PVS: Partner violence screen
CVI: Content validity index
CI: Cognitive interviews
I-CVI: Item content validity index
S-CVI: Scale content validity index
S-CVI/UA: Scale content validity index/universal agreement
S-CVI/Ave: Scale content validity index–average
ICC: Intraclass correlation coefficient
CI: Confidence intervals
SPSS: Statistical package for the social science
