# Supplementary material for: The questions on violence (FOV) tool for interpersonal violence inquiry in Swedish healthcare settings – evaluation of content validity, face validity and test-retest reliability
Source: BMC Health Serv Res. 2024 Oct 16;24:1240. doi: 10.1186/s12913-024-11708-3 (PMC11481745; doi:10.1186/s12913-024-11708-3)
Supplement: Supplementary file 1 — Supplementary Material 1. [file 12913_2024_11708_MOESM1_ESM.docx]

Supplementary file:

**Interview Guide Cognitive Interviews**

What is your year of birth?

What is your gender?

**Read the questions aloud in the order they appear in the questionnaire and ask the following questions in connection to each question in the questionnaire:**

- How do you interpret the question?
- What do you think when you read the question?
- Is the question clear?
- What does the question lack for it to be clear?

**General concluding questions:**

- What is your view of the vignette?
- How do you perceive the concept of ‘in close relationships’?
- Which of the headlines ‘physical violence’, ‘psychological/emotional violence’ and ‘sexual violence’ would you apply to questions 1–3?

**Additional questions:**

- What is missing for it to be clear?
- What is difficult to understand?
